# Spatial and temporal variation and hotspot detection of kala-azar disease in Vaishali district (Bihar), India

**DOI:** 10.1186/1471-2334-13-64

**Published:** 2013-02-02

**Authors:** Gouri Sankar Bhunia, Shreekant Kesari, Nandini Chatterjee, Vijay Kumar, Pradeep Das

**Affiliations:** 1 Department of Vector Biology and Control, Rajendra Memorial Research Institute of Medical Sciences (ICMR), Agamkuan, Patna 800007, Bihar, India; 2 Department of Geography, Presidency University, 86/1College Street, Kolkata 700073, West Bengal, India

**Keywords:** Kala-azar, Spatial statistics, Spatio-temporal, Hotspot

## Abstract

**Background:**

An improved understanding in transmission variation of kala-azar is fundamental to conduct surveillance and implementing disease prevention strategies. This study investigated the spatio-temporal patterns and hotspot detection for reporting kala-azar cases in Vaishali district based on spatial statistical analysis.

**Methods:**

Epidemiological data from the study area during 2007–2011 was used to examine the dynamic space-time pattern of kala-azar outbreaks, and all cases were geocoded at a village level. Spatial smoothing was applied to reduce random noise in the data. Inverse distance weighting (IDW) is used to interpolate and predict the pattern of VL cases distribution across the district. Moran’s *I* Index (Moran’s *I*) statistics was used to evaluate autocorrelation in kala-azar spatial distribution and test how villages were clustered or dispersed in space. Getis-Ord *G*_i_^*^(d) was used to identify the hotspot and cold spot areas within the study site.

**Results:**

Mapping kala-azar cases or incidences reflects the spatial heterogeneity in the incidence rate of kala-azar affected villages in Vaishali district. Kala-azar incidence rate map showed most of the highest endemic villages were located in southern, eastern and northwestern part of the district; in the middle part of the district generally show the medium occurrence of VL. There was a significant positive spatial autocorrelation of kala-azar incidences for five consecutive years, with Moran’s *I* statistic ranging from 0.04-0.17 (*P* <0.01). The results revealed spatially clustered patterns with significant differences by village. The hotspots showed the spatial trend of kala-azar diffusion (*P* < 0.01).

**Conclusions:**

The results pointed to the usefulness of spatial statistical approach to improve our understanding the spatio-temporal dynamics and control of kala-azar. The study also showed the north-western and southern part of Vaishali district is most likely endemic cluster region. To employ exact and geographically suitable risk-reduction programmes, apply of such spatial analysis tools should suit a vital constituent in epidemiology research and risk evaluation of kala-azar.

## Background

Kala-azar or visceral leishmaniais (VL) is one of the leading causes of morbidity and mortality in Bihar, India [1,2]. The disease is transmitted to the humans mainly by the vector, *Phlebotmus argentipes*[3]. The majority of VL (>90%) occurs in only six countries: Bangladesh, India, Nepal, Sudan, Ethiopia and Brazil [4]. In the Indian subcontinent, about 200 million people are estimated to be at risk of developing VL; this region harbors an estimated 67% of the global VL disease burden. The Bihar state only has captured almost 50% cases out of total cases in Indian sub-continent [5]. There is evidence that the disease are persisting at Vaishali district (Bihar), in India for many years [6], while others are more sporadic and persists for a short period of time only [7-9]. Vaishali district is the most seriously VL affected area in Bihar, with the highest number of kala-azar cases during the period from 2007 – 2011 (State Health Society report, Bihar, India). 12,200 cases and 24 deaths of kala-azar were recorded through active and passive surveillance system in 2007–2011 from the district only. The district accounted for >5 percent of total kala-azar cases reported from Bihar, and disease transmission in the district appeared the major focus fueling a sustained epidemic. The incidence of kala-azar shows high variability within the district.

For a vector-borne disease, it is important to recognize the spatial and temporal characteristics of its transmission. Geographic information technology (GIS) has a vital role in surveillance and control of the vector-borne diseases as it is promising to scrutinize factors associated with the disease through the geo-coding processes [10]. In the present study, we used several techniques under the umbrella of GIS. The application of GIS with spatial statistics, including spatial filtering (smoothing) and cluster analysis, pertained to the other diseases, where it is often used to investigate and more clearly exhibit the spatial patterns of disease [11-14]. On the other hand, the detection and enumeration of spatial heterogeneity in disease prevalence across a geographical area offer extent for targeting deterrence and treatment interventions at high-prevalence or high-risk areas [15,16]. Spatial statistics are the most useful tool for describing and analyzing how various geographical events occur [17].

GIS can not only give an avenue to expand our understanding of the distribution pattern of disease, but also assist public health officials to be in touch with public and policy makers about multifaceted information in a simply implicit configure. Hence, in this study, we aimed to check the spatio-temporal patterns of kala-azar disease in Vaishali district of Bihar, India using GIS tool and spatial statistical analysis.

## Methods

### Study area

**Figure 1:**
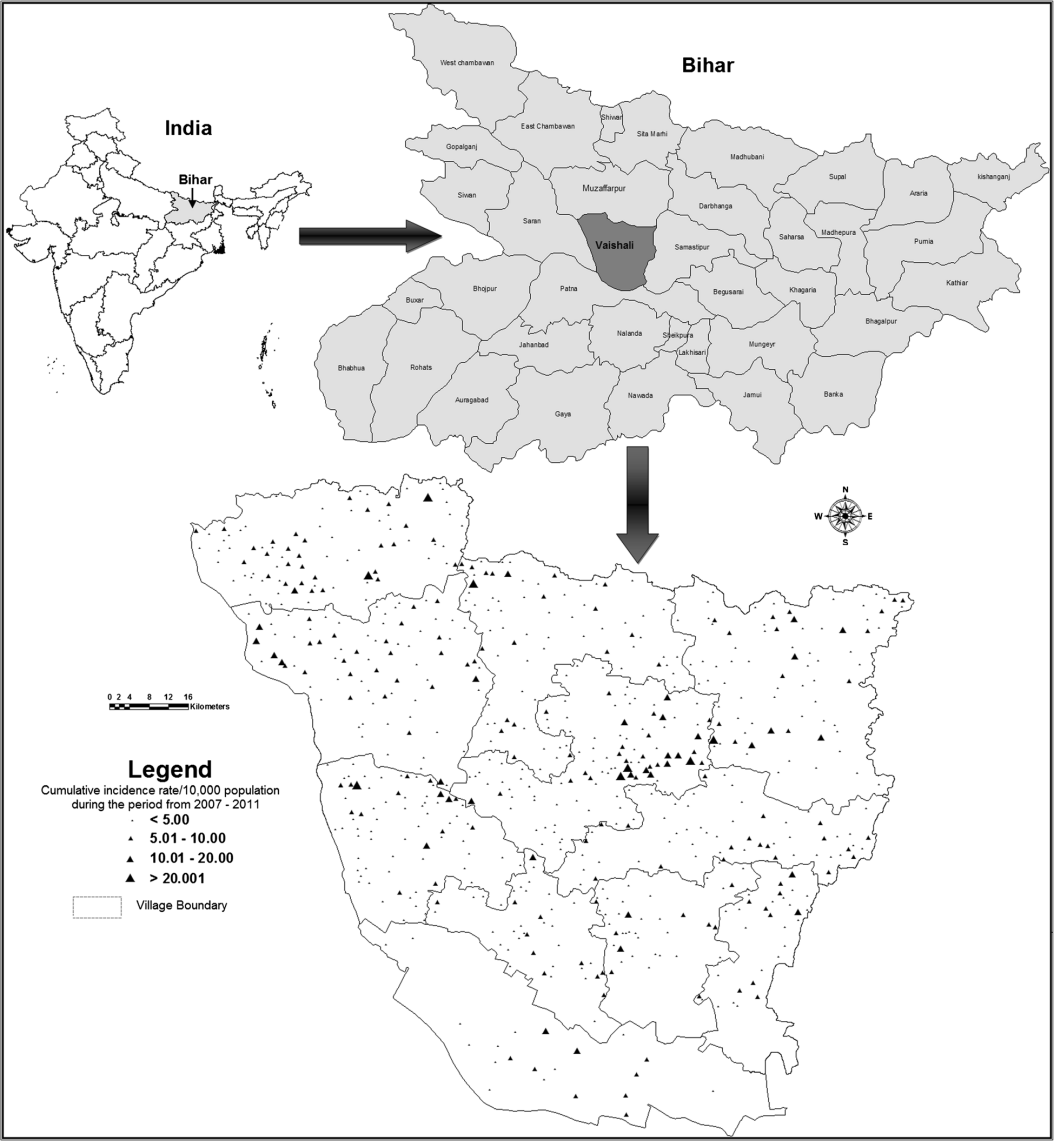
Location map of the study area.

An area covering 3173 km^2^ with a total population of 3,495,249 lying between 26.22°N – 25.55°N latitude and 84.53°E - 85.46°E longitude in North Bihar, India was selected for the main study (Figure 1). The climate of this region is humid tropical, with an estimated annual rainfall of 1,200-1,400 mm. According to weather station readings, daily mean minimum temperatures are 36.25°C in the summer, 33.5°C in the rainy, and 21.7°C in the winter season. All these conditions favour spread of this disease.

### Data collection and management

Kala-azar cases data reported in years 2007 to 2011 was used in this study. The data were obtained from the State Health Society (SHS), Bihar (India) and the Public Health Centers (PHCs). However, the results of the analysis were performed based on the secondary data, collected through the State and District Health Offices. In this study, location data and population data for 1741 villages of the district were collected from the department of Census of Bihar, India. For conducting a GIS-based analysis of the spatial distribution of kala-azar, the village-level point and polygon layer was generated based on the administrative boundary map (Scale-1:25,000) obtained from Land Revenue Office, Patna. All kala-azar cases were geo-coded and matched to the village-level layers of polygon and point by administrative code using the software ArcGIS 9.1 (ESRI Inc., Redlands, CA, USA).

### Measures of disease of occurrence

For the purpose of this study an average annual incidence rate was calculated for the period of 2007–2011. The numerator of five years cumulative VL cases were the sum of all kala-azar cases recorded during 2007–2011, and the denominator was the average number of population at risk during this period.

### Spatial filtering and smoothening

Data from all the kala-azar reported cases have been geo-coded using village location from the address of the patients. Spatial filtering can involve smoothing or sharpening the data of interest. The spatial smoothing was performed to reduce random noise in the data that comes from the high variance characteristic of small populations or small case numbers [18]. Voronoi statistics was opted to estimate the local smoothing and variation. In the present study, local smoothing procedure was used which includes the effects of spatial variability by using information from bordering the geographic area of neighbouring villages [19]. The smoothed incidence was computed from the total number of cases per 10,000 at each village divided by the total number of people at risk within the village, which was specified using a spatial weights file including the village. Specifically, spatial weights we used, is based on the inverse geographical distance between the centroids of the kala-azar affected and non-affected villages. Spatial weights are defined based on the 1 km distance between the centroids of neighboring villages. The value ‘1’ is used if the distance between the centroids of neighboring villages is within the specified distance (*e.g.*, 1 km); otherwise ‘0’ is incorporated in the weight file. Based on the annual average incidence, all villages were grouped into four categories: non-endemic village, low endemic village with annualized average incidence between 0 and 5 per 100,000, medium endemic area with the incidence between 5 and 30 per 100,000, and high endemic area with the incidence over 30 per 100,000. The technique of producing a smoothed map of disease rates allows for the display of data at a village level while preserving the stability of the estimated disease rates.

### Inverse distance weighting (IDW) interpolation

We used the IDW interpolation method to produce predictions of incidence rates of kala-azar cases across the whole district. This is because mapping the spatial distribution of kala-azar disease and potential risk areas requires, producing “bulls eyes” around data locations. The IDW interpolation technique is commonly used in GIS programme for producing surfaces using interpolation of scatter point and has been employed in other analysis of vector borne disease [20-22]. IDW is used to interpolate and predict the pattern of VL cases distribution across the district. The principle of IDW method is to consign more weights to nearby points rather than to distant points [23]. Since the IDW method is an exact method and is more accurate one, as it is sustain the entire probability distribution of incidence values, for which we have reliable data to support [24].

IDW weights the contribution of each input point by a normalized inverse of the distance from the control point to the interpolated point. The IDW interpolation method assumes that each input point has local influence that decreases with distance [25].

### Spatial autocorrelation

Spatial autocorrelation analysis was performed on the incidence rates of kala-azar to test whether the cases were distributed randomly over space and, if not, to evaluate any identified spatial disease clusters for statistical significance [26]. The Moran’s *I* Index (Moran’s *I*) statistics [27] was used to evaluate autocorrelation in kala-azar spatial distribution and test whether villages with high how (or low) incidence rates. The indices were evaluated by simulation, and considering the original location of the villages [28]. The value of Moran’s *I* range from −1 to +1: a value close to ‘0’ indicates spatial randomness while a positive value indicates positive spatial autocorrelation and vice-versa. We also computed Z-score and *p-*value associated with Moran’s *I*, indicates the likelihood that point pattern could be a result of random chance.

### Cluster-outlier analysis

Moran’s *I*, either general or local, can only detect the presence of the clustering of similar values. The Cluster-outlier (CO) type field distinguishes between a statistically significant (*P*<0.01) cluster of high values (High-High), cluster of low values (Low-Low), outlier in which a high value is surround primarily by low values (High-Low), and outlier in which a low value is surrounded primarily by high values (Low-High). A positive value for ‘*I*’ indicates that the feature is surrounded by features with similar values. Such a feature is part of a cluster. A negative value for ‘*I*’ indicates that the feature is surrounded by features with dissimilar values. Such a feature is an outlier. The Local Moran’s index can only be interpreted within the context of the computed Z-score or *p*-value. 99% significance level (*P* <0.01) was used to indicate significant clusters of local autocorrelation.

### Hotspot detection and analysis

Hotspot is defined as a condition indicating some form of clustering in a spatial distribution [29]. This has led to use of the Getis-Ord *G*_i_^*^(d), which can separate clusters of high values from cluster of low values [30,31]. Moreover, clusters of cases that occur randomly can also have an influence on the spread of an infectious disease. The local *G*_i_^*^(d) statistics is useful for determining the spatial dependence of neighbouring observations [32-34]. The result expresses the Z-score and *p*-value of the calculated *G*_i_^*^(d), in comparison with the normal distribution of the statistics calculated by simulation [35]. These values represent the statistical significance of the spatial clustering of values, given the conceptualization of spatial relationships and the scale of analysis (distance parameter). In this study, adjacency is defined using Thiessen polygon continuity weight file which has been constructed based on villages that share common vertices. The output from *G*_i_^*^(d) statistic identifies spatial clusters of high values (hotspots) and spatial clusters of low values (cold spots).

## Results

### Spatial analysis

Mapping kala-azar cases or incidences reflects the spatial heterogeneity in the incidence rate of kala-azar affected villages in Vaishali district. The high and/or low VL affected villages are not distributed uniformly in the study area, but they are strongly clustered in some particular parts across the district.

The highest adjusted incidence per 10,000 inhabitants was seen in 2008, while the lowest was in 2007. The map showed that the disease occurred everywhere in the district, mostly in villages in the eastern and northwestern part of the district. Smoothing provided a clear picture of the areas of kala-azar risks. The map for 2007 shows higher rates in the eastern and northern regions. The map for 2008 shows higher rates in the eastern and northwestern and in some tracts located in the southern regions were also more heavily affected. In 2009, there was a slight decrease in incidence rate involving in northeastern and middle regions of the district. However, in 2010, there was a slight increase in incidence involving peripheral tracts south, eastern, and northwestern regions. In 2011, the situation persisted into eastern regions, and a few villages in the northwestern regions.

**Figure 2:**
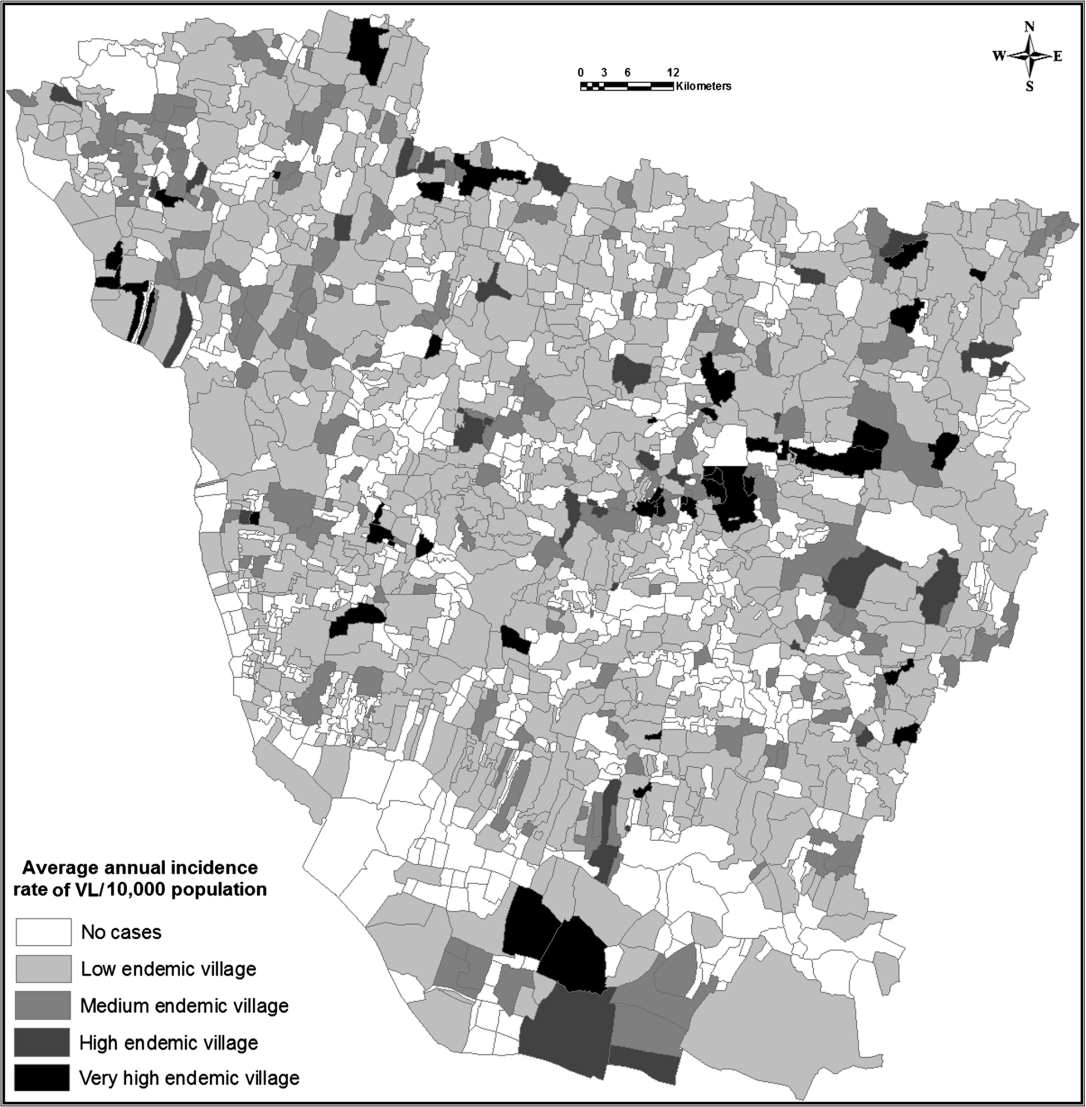
Map showing the average annual incidence rate of kala-azar from the period of 2007–2011.

In our analysis, a kala-azar incidence rate map was built from the cumulative number of cases for the period 2007–2011 (Figure 2), and the analysis result showed that kala-azar cases were spread all around the district, showing a very high endemic village in ‘dark black’ colour, high endemic village in ‘light black’ colour, medium endemic village with light ‘dark grey’ colour, low endemic village with ‘light grey’ colour and the blank area with no kala-azar cases. However, the results of the analysis showed most of the highest endemic villages were located in southern, eastern and northwestern part of the district; in the middle part of the district generally show the medium occurrence of VL. Nearly south and southwestern part of the district showed less incidence rate in last 5 years.

**Figure 3:**
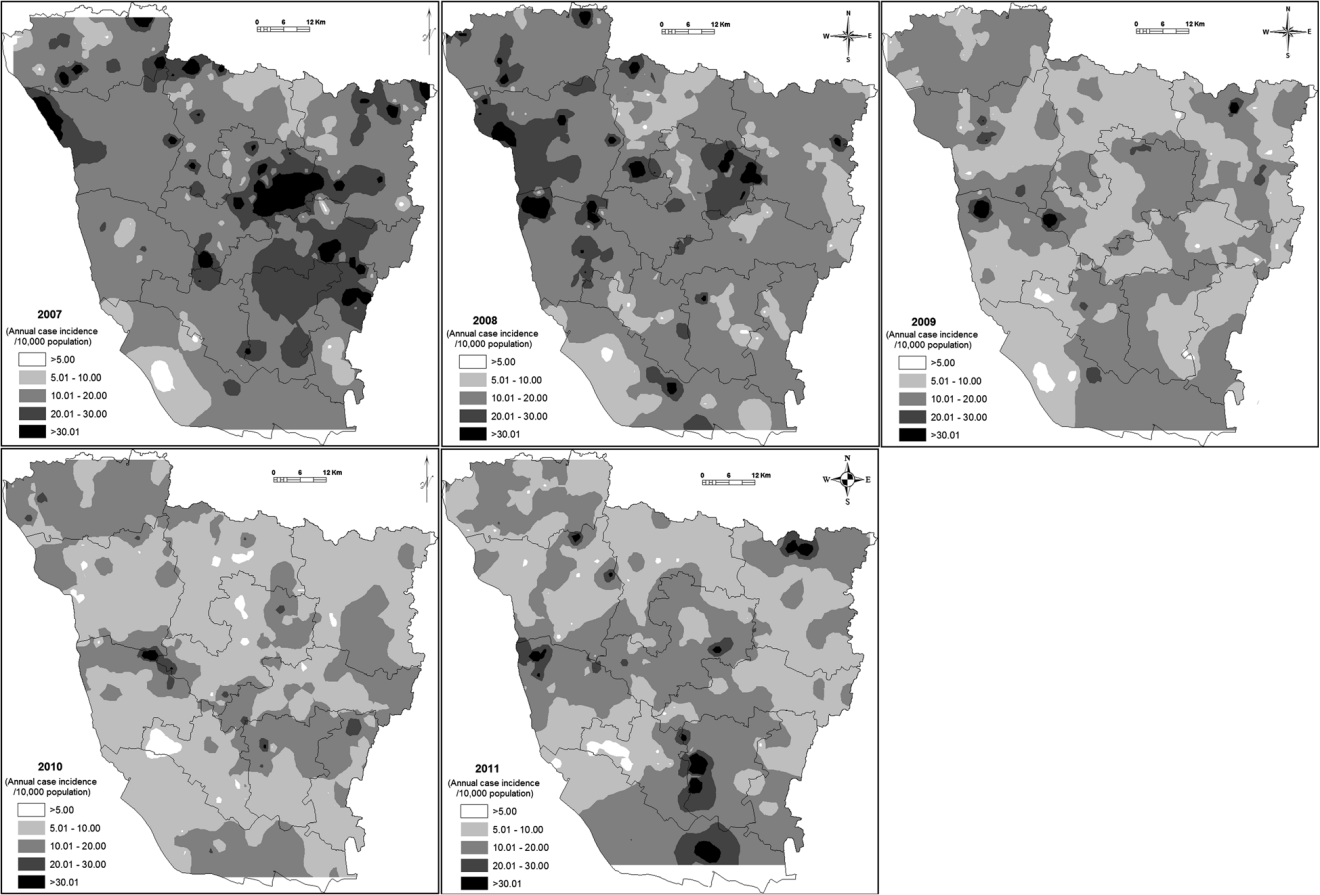
Maps showing the inverse distance weighting interpolation incidence rates of kala-azar disease over different years.

Figure 3 depicts the spatial distribution of interpolated estimates of incidence rates of kala-azar disease in the five successive years using IDW method, during 2007–2011. The intensity of gray levels is directly related to the intensity of VL occurrence in the study area, so the darker the area, the greater the risk of VL. It visually confirms that the incidence rates of kala-azar disease varied geographically across the district. In 2007 (Figure 3), we observed high-risk VL foci were found in eastern and some part of the northwestern part of the district. In 2008, the risk decreased in the eastern part, but there was a medium-risk VL foci in western regions. In 2009 and 2010, there was also an important reduction in VL intensity within the entire area. However, in 2011, a new focus was found in the southern and northeastern regions.

### Spatial autocorrelation of kala-azar distribution

The spatial autocorrelation analysis with Moran’s *I* Index showed that the spatial distribution of kala-azar incidence per 10, 000 inhabitants were clustered for all the years (2007–2011) and the results were highly significant (Table 1). The cases distribution in the year 2007, produces a Moran’s *I* highest value of 0.17 suggests that the likelihood of the pattern being a result of random chance is less than one percent. Conversely, the lowest of Moran’s *I* values were confirmed 0.04 in the year 2010. We can therefore bring to a close that the adjacent villages tend to have a similar kala-azar distribution, either high or low. However, the values of Moran’s *I* were of small magnitude, suggesting that clustering of VL probably occur only in small regions and/or scatter across the district. This information is a major finding to suggest public health departments that kala-azar is occurring in the cluster and not spread uniformly or randomly throughout the district. These locations may be considered as hot spots for future strategy to control.

**Table 1:** **Spatial autocorrelation estimation of different years using Moran’s *I *Index**

**Year**	**Observed**	**Expected**	**variance**	**Z-score**	**P-value**	**Pattern**
2007	0.165280	−0.00057	0.00020	11.6496	<0.01	Clustered
2008	0.099660	−0.00057	0.000199	7.100282	<0.01	Clustered
2009	0.070901	−0.000575	0.000196	5.101377	<0.01	Clustered
2010	0.044744	−0.000575	0.000045	4.120251	<0.01	Clustered
2011	0.057347	−0.000575	0.000198	6.677457	<0.01	Clustered

### High/low clustering analysis of kala-azar distribution

The high/low clustering tool is an inferential statistic, which means that the results of the analysis are interpreted within the context of a null hypothesis. Table 2 shows the clustering pattern kala-azar distribution with the incidence of cases per 10,000 inhabitants. The result of the analysis of G statistics showed kala-azar is highly clustered in the district. In this analysis *p-value* gives the probability that the observed spatial pattern was generated by some random process. When the *p-value* is very small, it means it is very unlikely (small probability) that the observed spatial pattern is the result of random processes, so the null hypothesis can be rejected. A high Z-score and small *p-value* for the VL affected villages indicates a spatial clustering of high values. The values of Z-score associated with G statistics showed that the intensity of clustering decreased in 2007–2011. The highest of G statistics values were confirmed 0.009 in the year 2007, while lowest was observed in 2010.

**Table 2:** Analysis of high/low clustering for different years using Getis-Ord Gi* statistic

**Year**	**Observed**	**Expected**	**variance**	**Z-score**	**P-value**
2007	0.00963	0.00320	0.000017	12.2138	<0.01
2008	0.00554	0.00320	0.000002	7.3240	<0.01
2009	0.00512	0.00320	0.000003	5.2544	<0.01
2010	0.00422	0.00320	0.000002	3.3341	<0.01
2011	0.00523	0.00320	0.000004	4.846	<0.01

### Cluster and outlier analysis of kala-azar distribution

Aggregate of villages with lower or a higher incidence rate are easily detected in the local cluster analysis. Significant small clusters of high risk were spread around the entire district (High-High); however, results also showed no significant spatial auto-correlation or a pattern of clustering of low incidence rate (Low-Low) in Vaishali district during the study period. Detailed characteristics of each cluster (per year) are presented in Table 3. There are other identified clusters, all associated with *p*-*values* greater than 0.05, which are not presented in the results. The presence of extreme *p*-*values* only in this current study (lower than 0.01) show clearly that statistically significant clusters are identified.

**Table 3:** Cluster and outlier analysis for kala-azar disease in Vaishali district, 2007–2011

	**Cluster-outlier type**															
**Year**	**High-high**				**High-low**				**Low-high**				**Low-low**			
	No. of villages	LMiIndex	LMiZScore	*p-*value	No. of villages	LMiIndex	LMiZScore	*p-*value	No. of villages	LMiIndex	LMiZScore	*p-*value	No. of villages	LMiIndex	LMiZScore	*p-*value
2007	63	28.25	11.92	0.003	11	−6.91	−2.64	<0.01	8	−5.34	−2.52	0.02	-	-	-	-
2008	73	11.83	5.00	<0.01	4	−7.47	−2.77	0.01	8	−5.21	−2.39	0.02	-	-	-	-
2009	49	13.72	6.01	0.009	5	−10.22	−4.12	0.009	4	−6.04	−2.54	0.019	-	-	-	-
2010	45	10.61	4.70	0.006	18	−8.50	−3.51	0.013	5	−5.95	−2.45	0.018	-	-	-	-
2011	38	16.37	7.04	0.004	10	−7.49	−3.03	0.014	4	−4.82	−2.26	0.026	-	-	-	-
2007-2011	67	58.00	9.11	0.008	17	−21.03	−3.28	0.014	22	−17.39	−2.44	0.021	-	-	-	-

Note: High-High: Cluster of high values; High-Low: outlier in which a high value is surrounding primarily by low values; Low-High: outlier in which a low value is surrounded primarily by high values; Low-Low: cluster of low values.

### Hotspot analysis

**Figure 4:**
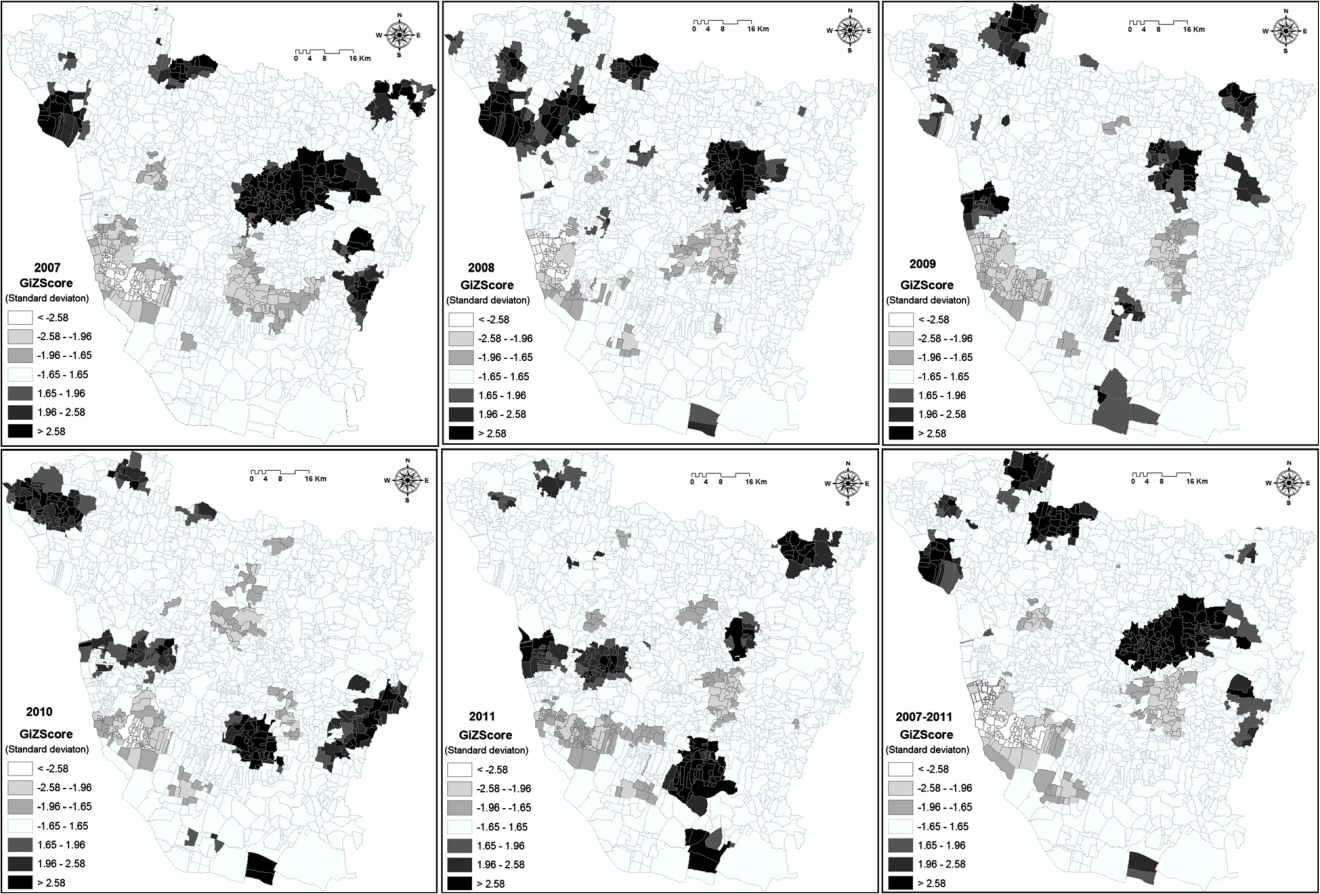
Maps showing the hotspot and cold spot over different years (2007–2011) in Vaishali district.

There were some outstanding spatial clusters of kala-azar covering specific locations. Each hotspot analysis of kala-azar incidence rate showed statistically significant hotspots (P<0.01). The analysis results show that the larger the Z-score is, the more intense the clustering of high values (hot spot); and the smaller the Z-score is, the more intense the clustering of low values (cold spot). In the map (Figure 4), darker areas indicate statistically significant hotspots, while, light areas represent significant cold spot areas. These maps show clear spatial patterns of kala-azar cases that were mostly spread east and north-west part of the district since 2007–2009. Some hotspot were also portrayed in the southern part of the district from 2010–2011. However, most of the cold spots were found in the southwest and middle of the study area, while, these are found in the urban and peri-urban region. The map for 2007 shows hotspot in the central and eastern region, in some tract in the north-western part; however, the cold spot was found in the lower central and south-western part of the district. The map for 2008 showed most of the hotspots were found in the north-western and central part of the district, and a small pocket was also observed in the southern part; however, the similar VL cold spot was also observed as in 2007. In 2009, there was a slight increase of VL hotspot areas in north-eastern and southern part; however, in 2010, there were no hotspot areas in north-eastern part and shifted to the southeast direction. In 2010, a cold spot was generated in the central part. In 2011, most of the hotspot areas shifted towards the southern direction, and a new focus of the hotspot was found in the north-east corner of the district. A kala-azar cases map was built from the cumulative number of cases for each year during the study period, and confirmed that kala-azar cases were spread all around the study area showing a hotspot with black colored points in the northwest and eastern part of the study area. This apparent cluster was due to a noticeable effect in the native village, where the spatial resolution of cases was lower.

## Discussion

This study reveals the spatial and temporal characteristics of kala-azar disease in Vaishali district of Bihar (India) using GIS tools and spatial statistical analysis, which allow for the quantification of the degree of clustering of VL infections. Such approaches have been used to investigate the spatial clustering of dengue [36], sleeping sickness [37], human granulocytic ehrlichiosis [38], haemorrhagic fever with renal syndrome [28], but their application to kala-azar has been less common, particularly in the Indian subcontinent. However, to our knowledge this is the first attempt to implement GIS mapping techniques to examine the distribution of kala-azar patterns in Vaishali district of Bihar (India). The present study has three major strengths. Firstly, this is the first study to examine the geographic variation of kala-azar disease across geopolitical boarders in kala-azar endemic areas of India using spatial statistics. This study lays a foundation for a further investigation of the spatial and temporal patterns and the risk factors of this disease. Secondly, the results of this study demonstrate that GIS mapping techniques may be used as a tool to quick display information and generate maps to highlight kala-azar disease risk prone areas for developing more effective control and prevention strategies. The maps could be used to suggest high risk areas where further investigation should be focused, to identify whether increased disease surveillance measures or possible control activities are warranted. Third, the kala-azar disease data used in this study are somewhat comprehensive, may be used for the national level. Finally, in this study, we aimed to examine the distribution patterns of kala-azar disease spatially and temporarily at the smallest geographical unit of Bihar, India.

An analysis of the spatial distribution or dependencies of the disease remains of the most important public health interest since the 1980s [39]. Using GIS and spatial statistics, the spatial pattern and distribution of confirmed kala-azar cases and increased risk regions in the highly endemic area were identified from 2007–2011. Spatial smoothing was used to estimate this underlying risk, which reduces differences in population size and in turns addressed variance instability and spurious outliers. The results obtained from smoothed VL incidence rates and inverse distance weighting (IDW) showed the high to low endemic villages and the progression of cases within the study area. Though, the earlier authors suggested that the performance of kriging was usually better that IDW to interpolate and predict the heterogeneous pattern of distribution [40,41]. In the IDW method, there is no assessment of the prediction errors, and no assumptions required by the data. However, the advantage of IDW is that it is intuitive and efficient, so for this kind of data IDW interpolation is recommended. In this study we were able to categorize clusters of high incidence rates in the northwestern, eastern and southern part of the district. However, in the southern part of the Vaishali district is surrounded by river Ganga and the north-western part of the district is flanked by the Gandak river that may retain surface moisture condition for the propagation of vector breeding [42]. Furthermore, the area has high population density, and is economically backward with main livelihoods option centered on agriculture and horticulture that may aids to the transmission of disease in this particular area [43,44]. An operational spinoff of this study is the indication that the use of the IDW to detect areas at highest risk of occurrence of VL may be useful for assigning VL surveillance and control measures. The identification of focal areas at greater risks can help define priority areas of specific interventions [45,46]. The evident existence of spatial clusters of high incidence and prevalence of VL advocates that the spatial distribution of the disease might be predisposed by environmental factors. And it has already been reported that transmissible disease with heterogeneous spatial distribution, targeted interventions tend to be the most effective [47].

The present results indicate that spatial statistics approach if carefully applied can play an important role in the recognition and analysis of the spatial structure of kala-azar epidemiology and control. The spatial association between cases is subject to the measure of geographical “closeness” or spatial proximity rather than a formal analysis [48]. The outbreak dynamic showed a clear non-random pattern of spreading from the first village to other villages in each year. Based on these analyses, investigators will be able to perceive clustering of areas with high detection rate of VL. Regarding the temporal variation of kala-azar disease, significant differences were noticeable across the district. Our results suggested that the annual incidence rates were fluctuated considerably, with the peak incidence rate in 2008. The reasons for spatial clustering of disease rates may put down in the heterogeneous allotment of essential factors such as crowding, social inequality, and access to health services or environmental characteristics [49-51].

Quantitative spatial analysis by using Moran’s *I* and Geary G statistics demonstrated that spatial distribution patterns of kala-azar cases were significantly clustered, and identified the kala-azar hotspots in Vaishali district. Spatial autocorrelation are valuable tools to study the spatial patterns over time. In this study, we found strong evidence of spatial autocorrelation of kala-azar disease across the district using Moran’s *I* statistics. The positive Moran’s *I* values indicate the spatial autocorrelation in disease distribution, indicated disease accumulated in some particular part of the area. However, in our analysis 0.17 is the highest value of Moran’s *I* statistics. The tracking analysis of the disease shows a cluster pattern in western and southern part of the study area. It may be due to the fact that the population of this region has lower economic indicators, subject to higher levels of social inequalities, which in turn through to increase susceptibility to VL disease. Aggregate of villages with lower or a higher incidence rate are easily detected in the local cluster analysis (significance level *P* = <0.01). Thus both the analysis confirmed that the spatial association of VL distribution occurred at the village level with significant high/low infection rate in the study sites. This means that the likelihood of one site becoming infested by *L donovani* increased when other sites in the peridomestic compound were infested. The analysis of risk estimates indicates small and significant low-high clusters (LH) surrounding the high-high (HH) cluster region in the southern, eastern and northwestern part of the district. Alternatively, cluster-outlier analysis did not show any clear spread pattern or trend during the study period. This could be due to the several factors. One possibility is that, VL or kala-azar transmission is going on unabatedly (*e.g.*, shimmering transmission). In addition, heterogeneity in the recovery time of infected individuals and behavioral changes, induced by the presence of cases, would alter the observed spatial pattern. This result suggests that the ideal conditions for establishments and maintenance of transmission are found in these places and that the pattern of VL occurrence is not static and disease may occasionally spread to other areas of the district.

We used *G*_i_^*^(d) statistic to identify the kala-azar hotspot based on the incidence rate/10,000 population. This technique provided a statistically robust and consistent method of detecting hotspot and cold spot areas within the district. This result suggested that the disease is spreading locally around foci, with waves of concentration diffusion process of hotspot and cold spot. Our findings revealed that cold spot was found in the urban and peri-urban region. This may be due to the several conditions that favour sandfly density, local climate, housing condition. Alternatively, poor neighborhoods tend to maintain some characteristics in the rural area, such as poor housing condition, lack of sanitation, bad living condition [52]. Another explanation would be that the heterogeneity in distribution of hotspot areas might simply reflects distortions provoked by public health surveillance system of different quality. Since this method may succeed to detect the local trends of VL distribution, which is probably the case now. This information could help to epidemiologist and health management professionals to mark out the areas of good habitat of vector and susceptible population for kala-azar. Consequently, the village locations were chosen as the best way to analyze the spatio-temporal patterns of outbreak dynamic over five consecutive years to study the temporal dynamics in space and time. However, in the present study, we identified patterns of dissimilarity of incidence of VL suggests heterogeneities in the underlying factors determining the transmission of *Leishmania donovani* or the detection of new hot spot areas between administrative villages.

## Conclusion

The result of this study show that epidemiological measures can be used to make out high and low risk areas more efficiently which aids in optimizing resources and minimizing VL cases. The present study provides public health planners with a more sophisticated tool to differentiate risk patterns of VL epidemic using spatial statistical approaches rather than relying on annual cumulative incidence alone, so that high-risk areas can be expansively recognized untimely in the epidemic based on their integrated spatial–temporal profiles. Some limitation of this study should also be highlighted, viz. despite the relatively high coverage of geocoding of VL cases; it is likely to have been worse in peripheral areas of the district. Second, whole analysis has been performed based on the area data. Despite these limitation, the study showed GIS and GIS-based spatial statistical techniques may provide an opportunity to clarify and quantify the epidemic situation of kala-azar within re-emerged epidemic areas, and lay a foundation to pursue future investigations into the environmental factors responsible for the increased disease risk. Spatio-temporal diffusion patterns and hotspot detection may offer useful information to sustain epidemiologist to control and predict kala-azar spread over critical hotspot areas only rather than for a whole region. Further, the methodology is based on notions on general principles of spatial statistics may employ the model to plan a strategy to control kala-azar by the information received on distribution and hotspots for various months or seasonally.

## Competing interests

The authors declare that they have no competing interests.

## Authors’ contributions

GSB, SK and NC participated in the article’s conceptualization and conducted the literature review, field data collection, analyzed and interpreted the compiled data and wrote the article. VK and PD participated in the article’s conceptualization and contributed to the analysis and interpretation of the results and helped write the article. All authors read and approved the final manuscript.

## Pre-publication history

The pre-publication history for this paper can be accessed here:

http://www.biomedcentral.com/1471-2334/13/64/prepub

## References

[B1] Guerin PJ Olliaro P Sundar S Boelaert M Croft SL Desjeux P Wasunna MK Bryceson AD Visceral leishmaniasis: current status of control, diagnosis, and treatment, and a proposed research and development agenda Lancet Infect Dis 2002 2 494 501 10.1016/S1473-3099(02)00347-X 12150849

[B2] Mubayi A Castillo-Chavez C Chowell G Kribs-Zaleta C Siddiqui NA Kumar N Das P Transmission dynamics and under reporting of Kala-azar in the Indian state of Bihar J Theor Biol 2010 262 177 185 10.1016/j.jtbi.2009.09.012 19769990

[B3] Swaminath CS Short HE Anderson LAP Transmission of Indian kala-azar to man by the bite of P. argentipes Indian J Med Res 1942 30 473 477 16789343

[B4] Chappuis F Sundar S Hailu A Ghalib H Rijal S Peeling RW Alvar J Boelaert M Visceral leishmaniasis: what are the needs for diagnosis, treatment and control? Nat Rev Microbiol 2007 5 873 882 17938629 10.1038/nrmicro1748

[B5] Joshi A Narain JP Prasittisuk C Bhatia R Hashim G Jorge A Banjara M Kroeger A Can visceral leishmaniasis be eliminated from Asia? J Vector Borne Dis 2008 45 105 111 18592839

[B6] Thakur CP Epidemiological, clinical and therapeutic features of Bihar kala-azar (including post kala-azar dermal leishmaniasis) Trans R Soc Trop Med Hyg 1984 78 3 391 398 10.1016/0035-9203(84)90131-7 6087515

[B7] Bora D Epidemiology of visceral leishmaniasis in India Natl Med J India 1999 12 2 62 68 10416321

[B8] Kesari S Bhunia GS Kumar V Jeyaram A Ranjan A Das P A comparative evaluation of endemic and non-endemic region of visceral leishmaniasis (Kala-azar) in India with ground survey and space technology Mem Inst Oswaldo Cruz, Rio de Janeiro 2011 106 5 515 523 10.1590/S0074-02762011000500001 21894370

[B9] Sudhakar S Srinivas T Palit A Kar SK Battacharya SK Mapping of risk prone areas of kala-azar (Visceral leishmaniasis) in parts of Bihar state, India: an RS and GIS approach J Vect Borne Dis 2006 43 115 122 17024860

[B10] Albrecht J Key concepts & Techniques in GIS 2007 Sage, Los Angeles

[B11] Bonet M Spiegel JM Ibarra AM Kouri G Pintre A Yassi A An integrated ecosystem approach for sustainable prevention and control of dengue in Central Havana Int J Occup Environ Health 2007 13 188 194 17718176 10.1179/oeh.2007.13.2.188

[B12] Frank C Fix A Peña C Strickland G Mapping Lyme disease for diagnostic and preventive decisions, Maryland Emerg Infect Dis 2002 8 427 429 10.3201/eid0804.000413 11971779 PMC2730243

[B13] Tran A Deparis X Dussart P Morvan J Rabarison P Remy F Polidori L Gardon J Dengue spatial and temporal patterns, French Guiana, 2001 Emerg Infect Dis 2004 10 615 621 10.3201/eid1004.030186 15200850 PMC3323097

[B14] Wu PC Lay JG Guo HR Lin CY Lung SC Su HJ Higher temperature and urbanization affect the spatial patterns of dengue fever transmission in subtropical Taiwan Sci Total Environ 2009 407 2224 2233 10.1016/j.scitotenv.2008.11.034 19157509

[B15] Hay SI Snow RW The malaria Atlas Project: developing global maps of malaria risk PLoS Med 2006 3 e473 10.1371/journal.pmed.0030473 17147467 PMC1762059

[B16] Nyarango PM Gebremeskel T Mebrahtu G Mufunda J Abdulmumini U Ogbamariam A Kosia A Gebremichael A Gunawardena D Ghebrat Y Okbaldet Y A steep decline of malaria morbidity and mortality trends in Eritrea between 2000 and 2004: the effect of combination of control methods Malar J 2006 5 33 10.1186/1475-2875-5-33 16635265 PMC1501031

[B17] Hay SI An overview of remote sensing and geodesy for epidemiology and public health application Adv Parasit 2000 47 1 35 10.1016/s0065-308x(00)47005-3 PMC3164799 10997203

[B18] Rushton R Lolonis P Exploratory spatial analysis of birth defect rates in an urban population Stat Med 1996 15 717 726 10.1002/(SICI)1097-0258(19960415)15:7/9<717::AID-SIM243>3.0.CO;2-0 9132899

[B19] Loh JM K-scan for anomaly detection in disease surveillance Environmetrics 2012 22 179 191

[B20] Hu W Tong S Mengersen K Oldenburg B Exploratory analysis of social and environmental factors associated with the incidence of Ross River viruses in Brisben, Australia Am J Trop Med Hyg 2007 76 814 819 17488897

[B21] Quinn HE Gatton MI Hall G Young M Ryan PA Analysis of Barmah forest disease activity in Queensland, Australia, 1993–2003: identification of a large, isolated outbreak of disease J Med Entomol 2005 42 882 890 10.1603/0022-2585(2005)042[0882:AOBFVD]2.0.CO;2 16363173

[B22] Woodruff RE Guest GS Garner MG Becker N Lindsay M Early warning of Ross River virus epidemics: combining surveillance data on climate and mosquitoes Epidemiology 2006 17 569 575 10.1097/01.ede.0000229467.92742.7b 16837824

[B23] Chang K Introduction to geographic Information Systems 2010 5 New York: Thomas D. Timp 327 340

[B24] Kheirandish S Liaghat M Azahar TM Gohari A Comparison of interpolation methods in prediction the pattern of basal stem rot diesease in palm oil plantation Geoinformatica - An International Journal (GIIJ) 2012 2 1 12 16

[B25] Fisher NI Lewis T Embleton BJJ Statistical analysis of spherical data 1987 Cambridge: Cambridge University Press

[B26] Kulldorff M Feuer EJ Freedman LS Breast cancer clusters in the Northeast United States: A geographic analysis Am J Epidemiol 1997 146 2 161 170 10.1093/oxfordjournals.aje.a009247 9230778

[B27] Boots BN Getis A Point Pattern Analysis Newbury Park 1998 Newbury Park, CA: Sage Publications

[B28] Fang L Yan L Liang S Vlas SJD Feng D Han X Zhao W Xu B Bian L Yang H Gong P Richardus JH Cao W Spatial analysis of hemorrhagic fever with renal syndrome in China BMC Infect Dis 2006 6 77 10.1186/1471-2334-6-77 16638156 PMC1471792

[B29] Osei FB Duker AA Spatial and demographic patterns of Cholera in Ashanti region-Ghana Int J Health Geogr 2008 7 44 10.1186/1476-072X-7-44 18700026 PMC2533654

[B30] Getis A Ord JK The analysis of spatial association by use of distance statistics Geographical Analysis 1992 24 189 206

[B31] Mitchell A The ESRI Guide to GIS Analysis 2005 2 Redlands, Calif: ESRI Press

[B32] Getis A Morrison AC Gray K Scott TW Characteristics of the spatial pattern of the dengue vector, Aedes aegypti, in Iquitos, Peru Am J Trop Med Hyg 2003 69 494 505 14695086

[B33] Hinman S Blackburn JK Curtis A Spatial and temporal structure of typhoid outbreaks in Washington D.C., 1906–1909: evaluating local clustering with the *i G statistic Int J Health Geogr 2006 5 13 10.1186/1476-072X-5-13 16566830 PMC1444925

[B34] Ord JK Getis A Local Spatial autocorrelations statistics: distributional issues and application Geogr Anal 1995 27 286 306

[B35] Feser E Sweeney S Renski H A descriptive analysis of discrete U.S. industrial complexes J Regional Sci 2005 45 395 419 10.1111/j.0022-4146.2005.00376.x

[B36] Jeefoo P Tripathi NK Souris M Spatio-Temporal Diffusion Pattern and Hotspot Detection of Dengue in Chachoengsao Province, Thailand Int J Environ Res Public Health 2011 8 51 74 21318014 10.3390/ijerph8010051 PMC3037060

[B37] Fèvre EM Coleman PG Odiit M Magona JW Welburn SC Woolhouse MEJ The origins of a new Trypanosoma brucei rhodesiense sleeping sickness outbreak in eastern Uganda Lancet 2001 358 625 628 10.1016/S0140-6736(01)05778-6 11530149

[B38] Chaput EK Meek JI Heimer R Spatial analysis of human granulocytic ehrlichiosis near Lyme, Connecticut Emerg Inf Dis 2002 8 943 948 10.3201/eid0809.020103 PMC2732548 12194771

[B39] Lawson AB Statistical Methods in Spatial Epidemiology 2001 New York: Wiley

[B40] Meng G Law J Thompson ME Small-scale health-related indicator acquisition using secondary data spatial interpolation Int J Health Geogr 2010 9 50 10.1186/1476-072X-9-50 20942935 PMC2964545

[B41] Yasrebi J Saffari M Fathi H Karimian N Moazallahi M Gazni R Evaluation and comparison of ordinary kriging and inverse distance weighting methods for prediction of spatial variability of some soil chemical parameters Res J Biol Sci 2009 4 1 93 102

[B42] Bhunia GS Kesari S Chatterjee N Pal DK Kumar V Ranjan A Das P Incidence of visceral leishmaniasis in the Vaishali district of Bihar, India: spatial patterns and role of inland water bodies Geospat Health 2011 5 205 215 21590671 10.4081/gh.2011.173

[B43] Arias JR Monteiro PS Zicker F The Reemergence of Visceral Leishmaniasis in Brazil Emerg Infect Dis 1996 2 2 145 146 10.3201/eid0202.960213 8903218 PMC2639817

[B44] Faucher B Gaudart J Faraut F Pomares C Mary C Marty P Piarroux R Heterogeneity of environments associated with transmission of visceral leishmaniasis in South-Eastern France and implication for control strategies PLoS Negl Trop Dis 2012 6 8 e1765 10.1371/journal.pntd.0001765 22880142 PMC3413717

[B45] Werneck GL Costa CH Walker AM David JR Wand M Maguire JH Multilevel modelling of the incidence of visceral leishmaniasis in Teresina, Brazil Epidemiol Infection 2007 135 195 201 10.1017/S0950268806006881 PMC2870576 16824254

[B46] Werneck GL Costa CH Walker AM David JR Wand M Maguire JH The urban spread of visceral leishmaniasis: Clues from spatial analysis Epidemiology 2002 13 364 367 10.1097/00001648-200205000-00020 11964941

[B47] Koopman JS Simon CP Riolo CP When to control epidemic infections by focusing on high-risk groups on high-risk groups Epidemiology 2005 16 621 627 10.1097/01.ede.0000172133.46385.18 16135937

[B48] White DM Blair CD Beaty BJ Molecular epidemiology of bluetongue virus in northern colorado Virus Res 2006 118 39 45 10.1016/j.virusres.2005.11.008 16337708

[B49] Fernández MS Salomón OD Cavia R Perez AA Acardi SA Guccione JD Lutzomyia longipalpis spatial distribution and association with environmental variables in an urban focus of visceral leishmaniasis, Misiones, Argentina Acta Trop 2010 114 2 81 87 10.1016/j.actatropica.2010.01.008 20096256

[B50] Neto JC Werneck GL Costa CHN Factors associated with the incidence of urban visceral leishmaniasis: an ecological study in Teresina, Piauí State, Brazil Cad Saúde Pública, Rio de Janeiro 2009 25 7 1543 1551 10.1590/s0102-311x2009000700012 19578575

[B51] Werneck GL Georeferenced data in epidemiologic research Ciência and Saúde Coletiva 2008 13 6 1753 1766 10.1590/s1413-81232008000600010 18833352

[B52] Kesari S Bhunia GS Kumar V Jeyaram A Ranjan A Das P Study of house-level risk factors associated in the transmission of Indian Kala-azar Parasites & Vectors 2010 3 94 10.1186/1756-3305-3-94 20937154 PMC2959033

